# Differential expression of the *FMRF* gene in adult and hatchling stellate ganglia of the squid *Loligo pealei*

**DOI:** 10.1242/bio.20136890

**Published:** 2013-12-10

**Authors:** J. Peter H. Burbach, Philip Grant, Anita J. C. G. M. Hellemons, Joseph A. Degiorgis, Ka Wan Li, Harish C. Pant

**Affiliations:** 1 Department of Translational Neuroscience, Brain Center Rudolf Magnus, University Medical Center Utrecht, 3584CG Utrecht, The Netherlands; 2 Marine Biological Laboratory, Woods Hole, MA 02543, USA; 3 Laboratory of Neurochemistry, National Institute of Neurological Disorders and Stroke, Bethesda, MD 20892, USA; 4 Department of Biology, Providence College, Providence, RI 02918, USA; 5 Department of Molecular and Cellular Neurobiology, Center for Neurogenomics and Cognitive Research, Neuroscience Campus Amsterdam, VU University, 1081 HV Amsterdam, The Netherlands

**Keywords:** FMRFamide, Cephalopods, Mollusk, Stellate ganglion, Giant axon, Prohormone processing

## Abstract

The giant fiber system of the squid *Loligo pealei* mediates the escape response and is an important neurobiological model. Here, we identified an abundant transcript in the stellate ganglion (SG) that encodes a FMRFamide precursor, and characterized FMRFamide and FI/LRF-amide peptides. To determine whether FMRFamide plays a role in the adult and hatchling giant fiber system, we studied the expression of the *Fmrf* gene and FMRFamide peptides. In stage 29 embryos and stage 30 hatchlings, *Ffmr* transcripts and FMRFamide peptide were low to undetectable in the SG, in contrast to groups of neurons intensely expressing the *Fmrf* gene in several brain lobes, including those that innervate the SG. In the adult SG the *Fmrf* gene was highly expressed, but the FMRFamide peptide was in low abundance. Intense staining for FMRFamide in the adult SG was confined to microneurons and fibers in the neuropil and to small fibers surrounding giant axons in stellar nerves. This shows that the *Fmrf* gene in the SG is strongly regulated post-hatching, and suggests that the FMRFamide precursor is incompletely processed in the adult SG. The data suggest that the SG only employs the *Fmrf* gene post-hatching and restricts the biosynthesis of FMRFamide, demonstrating that this peptide is not a major transmitter of the giant fiber system. This contrasts with brain lobes that engage FMRFamide embryonically as a regulatory peptide in multiple neuronal systems, including the afferent fibers that innervate the SG. The biological significance of these mechanisms may be to generate diversity within *Fmrf*-expressing systems in cephalopods.

## Introduction

FMRFamide is a biologically active tetrapeptide that has been implicated in the physiological control of various organ systems throughout the animal kingdom. FMRFamide was first identified in the bivalve mollusk *Macracallista nimbosa* by Price and Greenberg over 35 years ago (Price and Greenberg, 1977) and since then has appeared to be one of the most abundant regulatory peptides in both vertebrates and invertebrates with the notable exception of mammals where distantly related peptides are found (Fukusumi et al., 2006; Walker et al., 2009). In diverse species ranging from amphibians, crustaceans, insects, nematodes to hydroids, FMRFamide has been identified through immunological, proteomic and genetic approaches, and a multitude of biological functions has been assigned to this tetrapeptide, including its use as a neuropeptide in neural systems and as a hormone in peripheral organ systems (for reviews, see Li et al., 1999; Grimmelikhuijzen and Spencer, 1984; Santama and Benjamin, 2000; Fukusumi et al., 2006; Walker et al., 2009). In mollusks, FMRFamide has been shown to modulate neurotransmission and influence behavior (Baux et al., 1992; Cottrell et al., 1992; López-Vera et al., 2008). FMRFamide modulates contraction of somatic and visceral muscles (Lehman and Greenberg, 1987; Cottrell et al., 1983), mediates neural control of peripheral glands, acts on heart and kidney (Bulloch et al., 1988), and plays a role as a transmitter in chromatophore function in cephalopods (Loi et al., 1996; Loi and Tublitz, 2000).

FMRFamide is biosynthesized from a precursor protein together with structurally related peptides, the so-called FMRFamide-like peptides (FLPs). Most commonly, FLPs have the structure FXRF-amide, with X representing Met or Leu, or are extended at the N-terminus by additional amino acids. Comparison of phyla shows that though FMRFamide is the active peptide common to all investigated species, significant variation in FLPs exists, and that even greater differences exist in the architecture and structure of precursors, suggesting that they have diverged early in evolution (Taghert and Schneider, 1990; for a review, see Di Cosmo and Di Cristo, 2006). In mammals, there is no clear-cut ortholog of the FMRFamide precursor. The recently discovered *Rfrp* (RF-amide-related peptide) gene codes for peptides resembling FMRFamide (RF-amide-related peptides 1, 2, and 3) that have potent biological activities (Bechtold and Luckman, 2007). Additionally, two neuropeptide genes contain peptides with a C-terminal XRF-amide sequence: F8F-amide (morphine-modulating peptide) and neuropeptide FF encoded by the *Npff* gene, and alpha-neo-endorphin encoded by the *Enk* gene (Burbach, 2011a). These similarities suggest that these genes may have been derived from a common ancestral gene related to the *Fmrf* gene.

In cephalopods FMRFamide was first identified in *Octopus vulgaris* (octopus) (Martin and Voigt, 1987; for a review, see López-Vera et al., 2008) and studied physiologically in this species (for a review, see Di Cosmo and Di Cristo, 2006). The structural and neuroanatomical characteristics of cephalopod FMRFamide systems have been subjected to limited studies in *Sepia officinalis* (cuttlefish), *Loligo opalescence* (California squid), *Dosidicus gigas* (jumbo flying squid), and *Idiosepius notoidus* (pygmy squid) (Di Cosmo and Di Cristo, 1998; Di Cristo et al., 2002; Di Cristo et al., 2003; Di Cristo and Di Cosmo, 2007; Loi and Tublitz, 1997; Loi et al., 1996; Loi and Tublitz, 2000; Sweedler et al., 2000; Wollesen et al., 2008). It has been observed that the *Fmrf* gene and peptide in the CNS of the cephalopod *Idiosepius* are expressed embryonically (Wollesen et al., 2010). Only later FMRFamide peptide was seen in stellate ganglion (SG) neurites, after which other fiber tracts and lobes became immunoreactive.

The SG and its efferent giant fiber system drive mantle contraction for jet propulsion during the escape response. This system serves as an important neurobiological and physiological model by virtue of its unique size, particularly in the North Atlantic Long-finned or Woods Hole squid, *Loligo pealei* (Gilbert et al., 1990). The SG is part of the peripheral nervous system and is the third and last relay station in a chain of neurons that control this response. The first is a pair of giant neurons of the ventral magnocellular lobe (first order giant cells) (Young, 1939) of the central nervous system receiving sensory inputs. Through axo-axonal synapses, these contact axons of a second set of large motor neurons (second order giant cells) (Young, 1939) project through the pallial nerve to the SG. Within the SG, axons from small neurons of the giant fiber lobe (GFL) fuse to form the third order giant axons, each of which passes through the ganglion into the stellar nerves (Young, 1972). The stellar nerves consist of the giant axon surrounded by numerous smaller fibers from large motor neurons in the ganglionic walls that innervate mantle longitudinal muscles (Martin, 1965; Young, 1939; Young, 1972). Each stellar nerve also contains afferent sensory axons from the skin and smaller axons from the pallial nerves projecting from motor neurons in the posterior chromatophore lobe that do not synapse in the SG, but pass through to innervate chromatophore muscles of mantle and fin.

Because of the diverse functional roles of FMRFamide as neurotransmitter and/or modulator in mollusks and the appearance of FMRFamide in the SG of *ideosepius* (Wollesen et al., 2010), we aimed to determine if FMRFamide is a neuropeptide of the giant fiber system, and to compare the expression of the *Fmrf* gene and peptides in the SG in the adult and hatchling squid. In this study we find a single *Fmrf* gene that is differentially regulated in the SG of adult versus hatchling squid, and in the SG versus the central nervous system (CNS). The data show that FMRFamide is an early-expressed abundant neuropeptide in the CNS. The *Fmrf* gene appears not to be expressed in the SG of embryos and hatchlings, but it is highly expressed in adult SG giant fiber lobe neurons. Despite high gene expression, FMRFamide is not present as the tetrapeptide in the giant fiber system. It is restricted to *en passant* fibers and scattered microneurons of the SG. The data suggest that differential regulation of the *Fmrf* gene may serve to enhance diversity of FMRFamide peptides and their functions.

## Materials and Methods

### Animals

Live North Atlantic Long-finned squid (*Loligo pealei*) were obtained through the Marine Resources Center of the Marine Biological Laboratory, Woods Hole, MA, USA. To dissect the adult SG, we used the procedure described previously (Brown and Lasek, 1990). The adult squid were sacrificed by decapitation. Late embryos and hatchlings (stages 29 and 30), respectively, were obtained from squid egg fingers maintained in tanks with fresh running seawater at 20°C and staged according to Arnold (Arnold, 1965). Adult ganglia, embryos (stage 26–29) and hatchlings (stage 30) were harvested in seawater, fixed in 70% ethanol and stored at −20°C for *in situ* hybridization, or fixed in Bouins fixative or in 4% paraformaldehyde for paraffin embedding and immunohistochemistry. Six frozen adult ganglia and 3–5 frozen hatchlings were used for *in situ* hybridization. Six fixed adult SG, obtained from three squid and 3–5 each of Stages 29 and 30 fixed embryos were used for the FMRFamide immunohistochemistry.

### Construction and sequencing of cDNA libraries

SGs were dissected from adult squid and stored at −80°C. Poly(A+)RNA was isolated, size selected, and multiple libraries were made in the Lambda-Express vector by unidirectional cloning of cDNAs using kits of Stratagene (Santa Clara, CA, USA). Another library was custom-made. Libraries were plated and plaques were randomly picked and sequenced, either at small scale in a Beckman SEQ2000 or by the National Human Genome Research Institute-NIH, Bethesda, MD, USA. In total 22,689 high-quality EST sequences with an average length of 618 nucleotides were obtained, as described (DeGiorgis et al., 2011). Selected clones were sequenced from the 5′ ends.

### *In situ* hybridization

Mantle sections containing adult SGs, embryos, and hatchling squid were embedded in TissueTek and cryosectioned. *In situ* hybridization on 20 µm sections using DIG-labeled RNA probes was performed as described (Smidt et al., 2004). The probe used was a full-length *Fmrf* cDNA of *Loligo pealei*. Probes of squid *α-tubulin* and *collagen* were obtained by PCR.

### Immunohistochemistry

Dissected adult stellate ganglia, embryos, and hatchlings were fixed in Bouin's fixative or 4% paraformaldehyde, embedded in paraffin and 5 µm sagittal, horizontal, and coronal sections were used for immunocytochemistry as described previously (Grant et al., 1995). Rabbit anti-FMRFamide polyclonal antisera from Chemicon International (AB1917) and from ABCAM (ab10352) were used as primary antibodies. Both antisera were titered for optimal signal-to-background ratio and used at a final concentration of 1:750. If not specified otherwise, antiserum AB1917 (Chemicon International) was used. Immunoreactivity was detected by DAB-staining using the Vectastain Universal Elite ABC Kit (Pk-6200, Vector Laboratories, Burlingame, CA, USA). As controls for specificity, sections were treated with primary antisera pre-incubated with FMRFamide. Sections were counterstained in 0.2% methyl-green. In one experiment, hatchling sections were treated with a rabbit-derived polyclonal antibody to squid phospho-heavy neurofilament (NFH) (1:500). Separate sections were treated with hematoxyline and eosin (H&E) stain. Photomicrographs were taken with a Zeiss Axiomat microscope system and resulting images adjusted for white balance, brightness, contrast and color level distribution using Photoshop or the Gimp open source software packages.

### Silver staining

Bouin-fixed, paraffin embedded serial sections (10 µm) of hatchlings were stained as described previously (Gregory, 1980). Sections were impregnated in 2% protargol solution (pH 8.0) containing copper (1.3 gm/65 ml) for 12 hours, washed, then developed in 1% hydroquinone containing 2.5% sodium sulfite, washed, gold intensified, dehydrated, cleared and mounted in Permount.

### Proteomic analysis

Extracts of the SG and the optic lobe were separated by reverse phase HPLC and fractions subjected to mass spectrometry, essentially according to El Filali et al. (El Filali et al., 2006). Extracts, equivalent to half an optic lobe or two stellate ganglia, were homogenized in 2 ml of acid acetone (acetone:HCl:H_2_O = 40:1:6 v/v), vortexed and centrifuged at 16,000×g for 5 min. The supernatant was diluted 1:20 with 7 mM trifluoroacetic acid (TFA), and then loaded onto a 3 ml C18-Sepak column for pre-purification. The bound peptides were eluted from the Sepak column with 2 ml 60% acetonitrile/7 mM TFA, and then speedvac dried. Samples were redissolved in 250 µl 0.1%TFA, and 200 µl was injected into a HPLC-C18 reverse phase (2×250 mm) column eluted at 200 µl/min with increasing concentration of acetronitrile.

One µl of each HPLC fraction was spotted onto a MALDI plate, partially dried, and 0.5 µl MALDI matrix was added. MALDI MS and consecutively collision-induced dissociation MSMS with an inclusion mass of 599.3 Da corresponding to that of FMRFamide, was performed. MSMS were triggered when the signal to noise ratio of the peptide at MS1 was above 100. In total 307 MSMS analyses were performed on the SG sample, and 450 on the optic lobe sample. Procedures have been described previously (El Filali et al., 2006).

## Results

### Properties of the predicted FMRFamide precursor

Sequence analysis of randomly-picked clones from two cDNA libraries of the squid stellate ganglia resulted in the identification of a 1,692 nt transcript that encoded a FMRFamide precursor protein. This transcript was represented by 228 out of 22,662 sequenced clones (1.2%) and was amongst the most abundant cDNAs in the libraries (DeGiorgis et al., 2011). The nucleotide sequence was deposited in GenBank under accession number FJ205479. Comparison of all clones showed that the cDNAs represented a single transcript with few polymorphisms. No splice variants were detected.

**Fig. 1.**
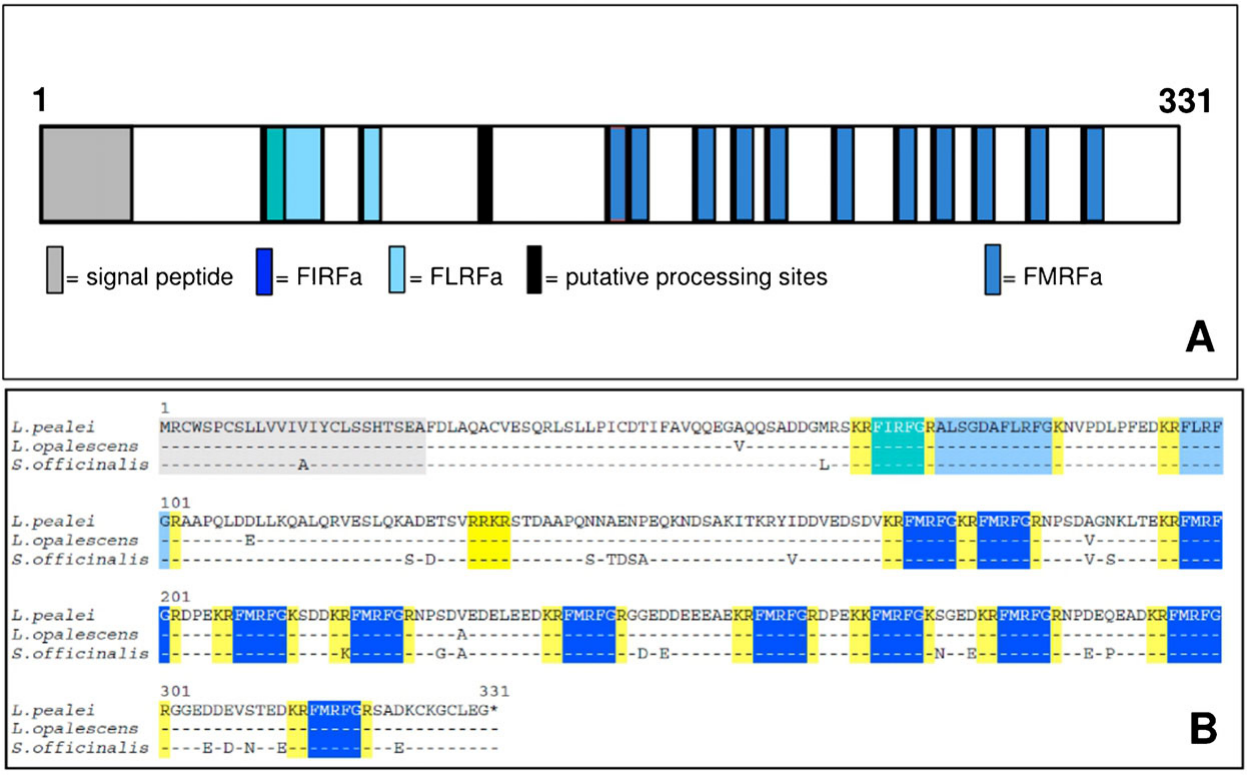
Structure and amino acid sequence of the FMRFamide precursor of the squid *Loligo pealei* (*L. pealei*) and comparison to related decapodiforme species *Loligo opalescens* (*L. opalescens*) and *Sepia officinalis* (*S. officinalis*). The amino acid sequence was deduced as the largest open reading frame from a 1692 nt transcript (GenBank accession number FJ205479.1). (A) The precursor contains eleven perfect FMRFG pentapeptide motifs (dark blue), one FIRFG sequence (green), and two FLMRFG motifs (light blue) of which one is extended at the N-terminus. The C-terminal glycine serves as substrate for amidation resulting in FMRFamide tetrapeptide. The motifs are flanked by pairs of basic amino acids (black) that are putative processing sites by prohormone convertase. The 25-amino acid signal peptide was predicted by SIG-Pred (Bradford, 2001) and SignalP (Nielsen et al., 1997) and is shown in grey. (B) Amino acid sequence of FMRFamide precursors of related cephalopods. Differences are shown. Amino acid sequences were translated from the nucleotide sequences entries AF303160.1 and Y11246.1.

The largest open reading predicted a 331 amino acids protein with a 25-amino acid signal peptide at the N-terminus according to the SIG-Pred (Bradford, 2001) and SignalP 3.0 algorithms (Nielsen et al., 1997). This protein encoded 11 copies of the amino acid motif KK/RFMRFGK/R (Fig. 1A). This motif consisted of the FMRF core sequence, C-terminally flanked by a glycine residue that serves as amide donor of the preceding phenylalanine, and flanked at both sides by basic amino acid residues that serve as cleavage sites for prohormone convertases. This motif predicted that end products of complete biosynthetic processing are multiple copies of the peptide FMRFamide. One copy of the FMRFamide related peptides FIRF-amide and one FLRF-amide copy were predicted as well as one longer form ALSGDAFLRF-amide (Fig. 1B).

Comparison with FMRFamide precursors of other cephalopod species showed similarity in organization and amino acid sequence to that of the related decapodiformes *Loligo opalesensce* (98% over 331 aa) and *Sepia officinalis* (92% over 331 aa) (Fig. 1B). However, the FMRFamide precursors of these cephalopod species are significantly different from those of other molluscan species such as the gastropods *Aplysia californica* (45.5% over 297 aa), *Lymnea stagnalis* (42.2% over 268 aa), and *Haliotis asinine* (45.2% over 361 aa) and the bivalve *Mytilus edulis* (43.6% over 381 aa) (data not shown). There was no significant similarity in protein architecture to FMRFamide precursors of other invertebrate and vertebrate species.

### Expression of the *Fmrf* gene in the adult SG

**Fig. 2.**
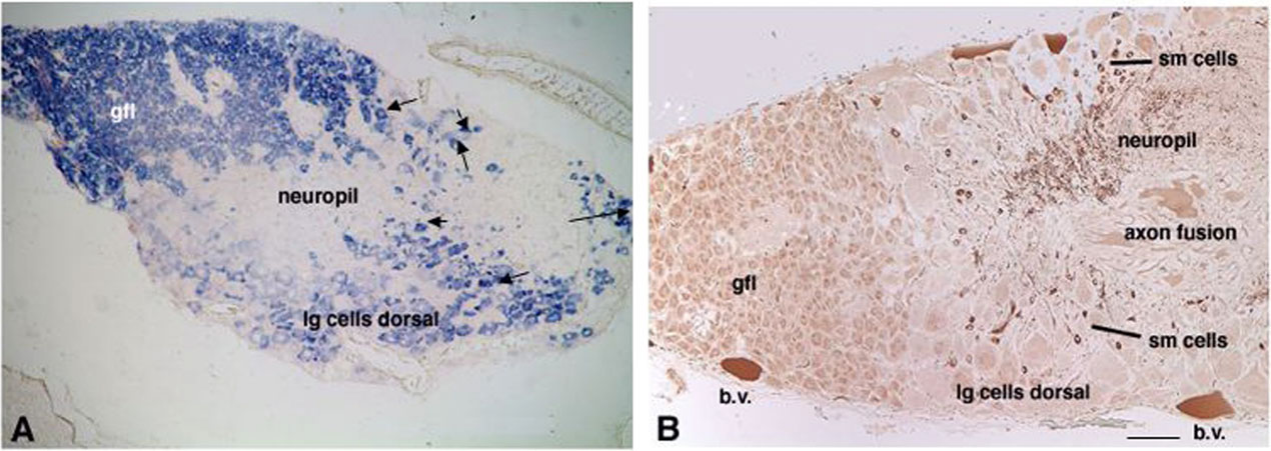
Expression of the *Fmrf* gene and FMRFamide-immunoreactivity in the adult SG. (A) Cryosections of the dorsal mantle containing the SG were hybridized to digoxigenin-labeled RNA probes to localize *Fmrf* mRNA. Expression was strong in neurons of the giant fiber lobe (gfl) and in small intraganglionic neurons (arrows). Large neurons in the dorsal and ventral walls displayed weaker expression. No expression was detectable in the neuropil and giant axon. (B) FMRFamide immunoreactivity in the adult SG. Low magnification of the ganglion showing immunoreactivity in small cells of the giant fiber lobe (gfl) and more robust staining in fibers of the neuropil. Small cells (sm cells) adjacent to neuropil also stain intensely. Large cells of the dorsal and ventral walls show low levels of expression while regions of axon fusion into 3^rd^ order giant fibers also exhibit low or negative expression. b.v. are blood vessels. Note low levels of expression are comparable to control sections pre-incubated with the FMRFamide. See Fig. 3B. Scale bar: 100 µm.

The observation that *Fmrf* clones were amongst the highest represented in the EST library suggested a high level of expression of this gene in the adult SG. Expression of the *Fmrf* gene in this ganglion was examined by *in situ* hybridization. DIG-labeled antisense RNA probes readily detected *Fmrf* transcripts in all neurons of the adult SG (Fig. 2A). No signals were obtained with a sense probe used as negative control. The smaller neurons of the GFL exhibited the most intense gene expression in a well-defined region of densely packed cells whereas the larger cells in dorsal and ventral sectors showed a mosaic of strong and weakly expressing neurons. However, not all neurons contained detectable levels of *Fmrf* mRNA. Small neurons scattered in the intraganglionic mass displayed a high signal of *Fmrf* mRNA. The neuropil in the intraganglionic mass did not display *Fmrf* expression (Fig. 2A). Comparison to the expression patterns of the *β-tubulin* gene, a neuronally expressed gene, and *collagen*, a non-neuronal gene, confirmed the neuronal nature of FMRFamide precursor expression (data not shown).

**Fig. 3.**
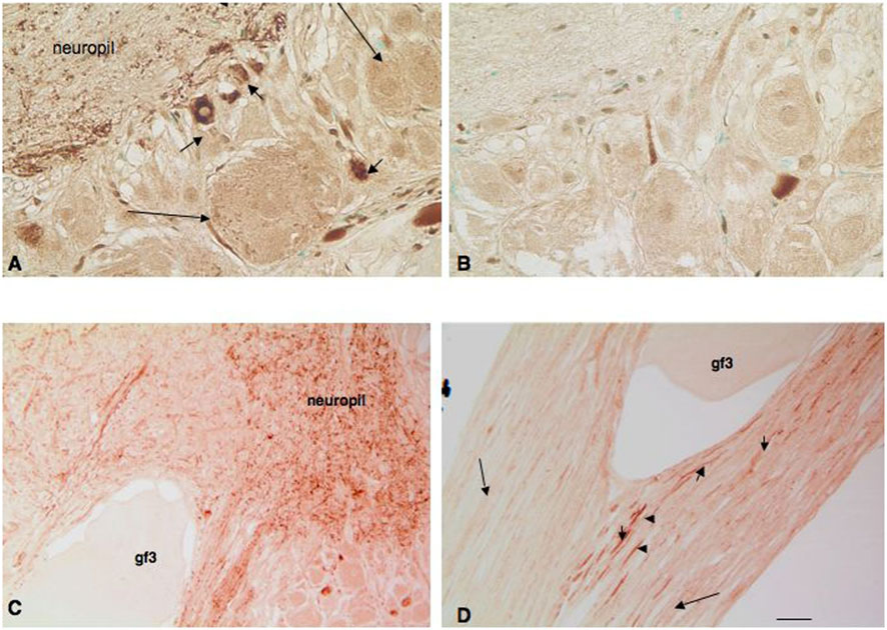
FMRFamide immunoreactivity in the adult SG and stellar nerves. (A) High magnification of the ganglion showing strong immunoreactivity in neuropil fibers and adjacent small cells (small arrows). Large arrows identify large cells. (B) An adjacent control section pre-absorbed with FMRFamide peptide exhibits no staining in neuropil fibers and small cells. Low level background staining persists, however, in large cells suggesting a modest level of cross reactivity with other proteins. (C) High magnification of the ganglion showing a giant axon (gf3) within a stellar nerve as it leaves the ganglion. No staining is seen in the giant axon whereas neuropil fibers and a few small cells are intensely stained. (D) A more distal section of the stellar nerve seen in panel C. Gf3 axon is unstained whereas small *en passant* fibers (small arrows) within the nerve are intensely reactive with FMRFamide. Large arrows identify larger axonal fibers from the SG expressing no FMRFamide immunoreactivity. Scale bar: 20 µm.

Immunohistochemistry with two different antisera against the FMRFamide peptide was used to detect and locate FMRFamide (Fig. 2B). Strong FMRFamide staining was only observed in scattered small neurons in the intraganglionic mass and in the neuropil (Fig. 3A,C). This staining could be eliminated by pre-absorption with FMRFamide (compare Fig. 3A with Fig. 3B). Surprisingly, the staining for FMRFamide of neurons in the GFL was weak, while this lobe displayed the strongest *in situ* labeling for *Fmrf* mRNA (Fig. 2A). A few large neurons also displayed weak FMRFamide staining, often detectable as punctate intracellular structures (Fig. 3A).

Examination of cross-sections containing the giant axon revealed weak FMRFamide immunopositivity in small stellar nerves surrounding the giant axon (Fig. 3D, lg arrows) while the giant axon (gf3) itself did not contain FMRFamide immunoreactivity (Fig. 3C,D). Robust staining of fibers within the stellate nerve was observed as the giant axon leaves the ganglion (Fig. 3C,D). In addition, small cells in the ganglionic mass were strongly FMRFamide positive (Fig. 3A, small arrows). Sections of stellar nerves showed immunoreactive fibers within and leaving the neuropil (Fig. 3C,D, arrow heads). These fine immunoreactive fibers may represent nerve fibers passing through the SG entering via the pallial nerve, described by Young (Young, 1939; Young, 1972).

The data revealed a notable mismatch between the high expression of the *Fmrf* gene and the low FMRMamide immunoreactivity in the giant fiber lobe. Since the antisera are directed against the amidated C-terminus of the tetrapeptide, and this is absent in the precursor, the weak staining of FMRFamide in this lobe and the absence of peptide immunoreactivity in the 3^rd^ order giant axons suggests that the level of FMRFamide peptide produced by these neurons is very low. One possible explanation could be an inefficient processing of FMRF precursor into FMRFamide. Therefore, the chemical nature of FMRF-peptides was investigated next.

### Proteomic analysis of stellate lobe extracts

**Fig. 4.**
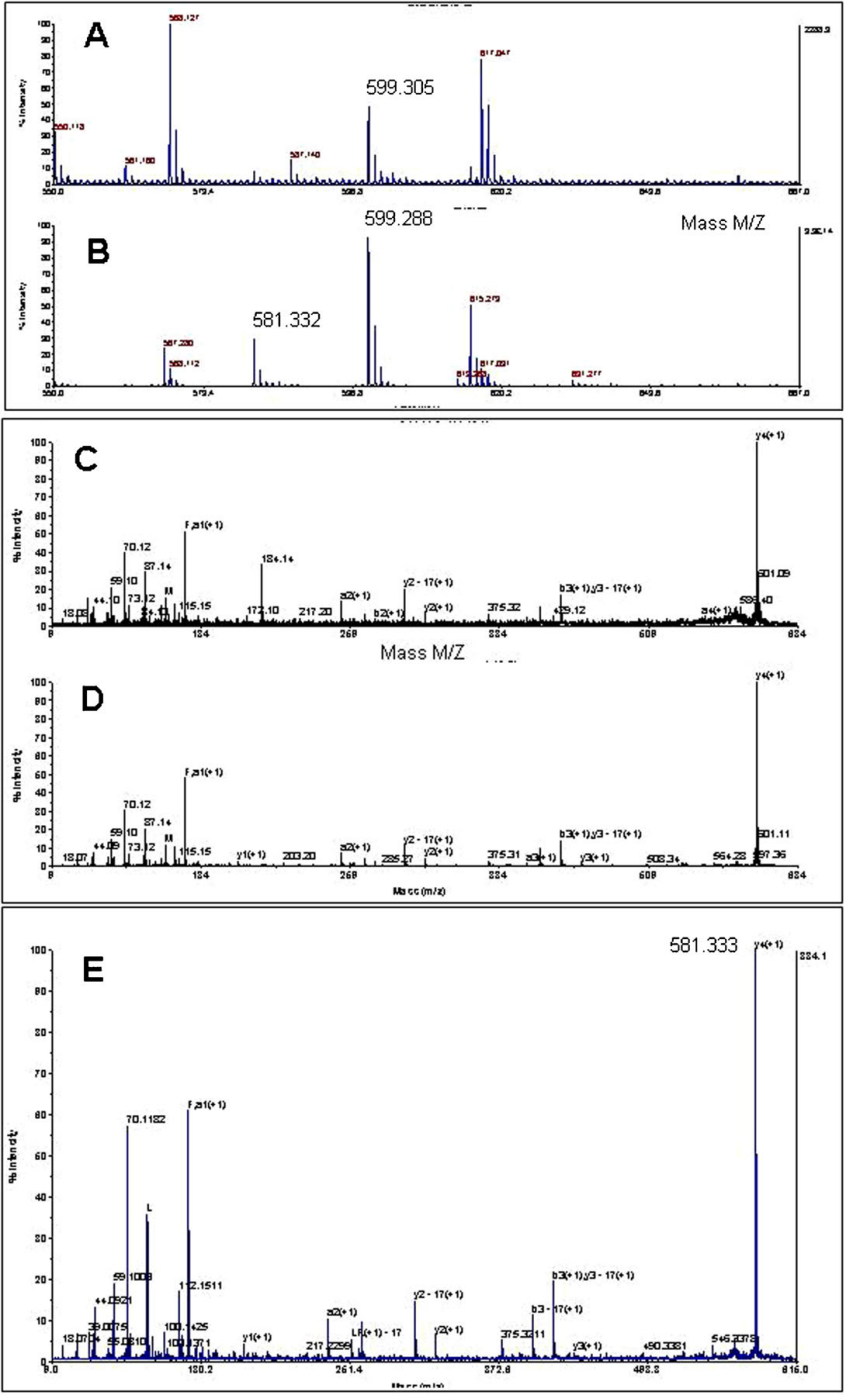
Proteomics analysis of peptide extracts of the SG and optic lobe. MALDI mass spectra of peptides were obtained after HPLC fractionation of extracts. (A,B) Partial spectra of mass spectrometric survey scans in MS1 in the mass range of 550 to 1000 Da containing peptides of the SG (A) or optic lobe (B). (C,D) Mass spectra of high energy collision-induced dissociation MSMS with an inclusion mass of 599.3 Da corresponding to that of FMRFamide from SG (C) or optic lobe (D). MSMS was triggered by a signal to noise ratio of the peptide at MS1 above 100. (E) Mass spectra of high energy collision-induced dissociation MSMS with an inclusion mass of 581.3 Da corresponding to that of FIRF-amide or FLRF-amide of molecules obtained from the optic lobe.

In order to examine the chemical nature of products of the FMRF precursor in the SG, peptide extracts were analyzed by mass spectrometry and compared to the optic lobe, which contains scattered but intensely staining FMRFamide immunoreactive neurons and fibers (data not shown). To this end, extracts of the SG and optic lobe were fractioned by HPLC and peptides analyzed by MALDI-MSMS (El Filali et al., 2006). Both extracts contained a peptide of 599.3 Da identical to the mass of FMRFamide (Fig. 4A,B). The signal intensity of the 599.3 Da peak from the optic lobe was 16 times stronger than that of the SG (Fig. 4A,B), indicating that the concentration of FMRFamide in the optic lobe extract was higher than that in the SG. Collision-induced dissociation (MSMS) of this 599.3 Da peptide from both optic lobe and stellate ganglia showed the characteristic fragmentation pattern of FMRFamide (Fig. 4C,D), demonstrating the identity of this tetrapeptide.

The FMRFamide-related peptide FLRF-amide or FIRF-amide (581.3 Da) was also identified in stellate and optic lobe extracts, again with a far higher concentration in optic lobe extracts than in SG extracts (Fig. 4A,B). The quantities of these peptides relative to FMRFamide corresponded to the precursor structure. The non-amidated form FMRFG (657 Da) was not detected above the noise level (Fig. 4A,B). These analyses demonstrated that FMRFamide was present in both tissues, but that the tissue concentration of FMRFamide was many-fold higher in the optic lobe than in the SG. The possibility that the SG would produce the non-amidated form of FMRFG was excluded. Therefore, despite the high levels of *Fmrf* mRNA levels in the SG, the content of FMRFamide was low, indicating that stellate neurons are inefficient in translating this mRNA, or that precursor processing as compared to optic lobe neurons is limited. The latter is a form of differential processing well known for other neuropeptide precursors (Burbach, 2011b).

Attempts to detect the FMRF precursor on Western blots by available FMRFamide antisera failed. These likely do not recognize the FMRF precursor, since they were directed against the amidated C-terminus of the peptide that is generated as a post-translational modification during precursor processing. This antigen is not present in the precursor protein.

### Expression of the *Fmrf* gene in SG of the late embryo and hatchling

**Fig. 5.**
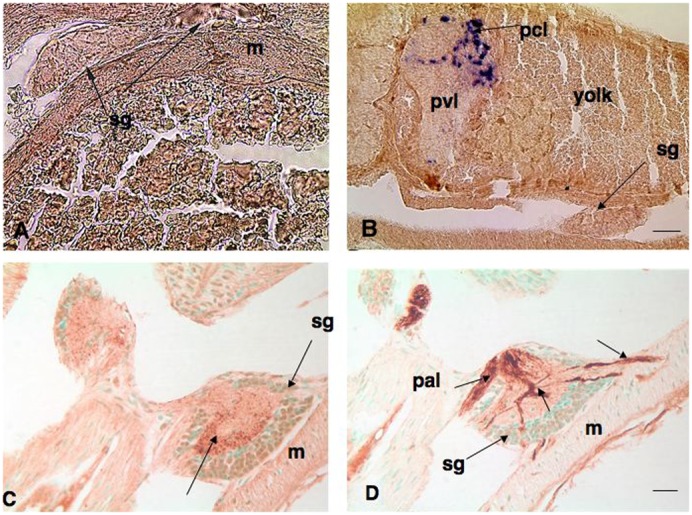
Expression of *Fmrf* transcripts and FMRFamide peptide in sections of stage 29 and 30 squid. (A) *In situ* hybridization of a coronal section of a stage 29 embryo with large yolk mass showing no expression of the *Fmrf* gene in stellate ganglia (arrows). (B) A sagittal section of a stage 29 embryo showing deeply staining cells in the post chromatophore lobe (pcl) in the dorsal region of the palliovisceral lobe (pvl). Here, too, the SG (sg) shows no expression of *Fmrf*. (C) A horizontal section of a stage 30 hatchling showing contrasting FMRFamide staining in the SG with FMRFamide staining in fibers of the central neuropil (arrows) surrounded by unlabeled cells. (D) A horizontal section of a hatchling immunoreactive with a squid-specific neurofilament antibody showing robust expression in nerve fibers and tracts associated with the SG (sg). Pallial nerve (pal) containing a 2^nd^ order giant fiber enters to synapse within neuropil, while 3^rd^ order giant axons (arrows) leave to innervate muscles in the mantle (m). Scale bars: 50 µm (A,B), 20 µm (C,D).

In sections of the SG of embryos (stage 29) and hatchlings (stage 30), it was not possible to accurately distinguish the presumptive GFL neurons from large neurons of the dorsal and ventral ganglion walls (Fig. 5). Embryos and hatchlings at these stages expressed the *Fmrf* gene and contained FMRFamide immunoreactivity in brain ganglia (Fig. 5). However, there was no expression of the *Fmrf* gene detectable by *in situ* hybridization in the SG in all embryos and hatchlings examined (Fig. 5A). On the other hand, immunocytochemistry showed very weak FMRFamide staining of neuronal cell bodies in the SG (Fig. 5C). Intense peptide expression, however, was seen in the central intraganglionic mass in the form of fine fibers. In contrast, the FMRFamide immunoreactivity in central brain ganglia (basal and palliovisceral lobes) was much more intense supporting the idea that FMRFamide was present at barely detectable levels in SG neurons. Nor could it be detected in embryonic 3^rd^ order giant axons leading to the mantle. When embryonic sections were incubated with an antibody specific for squid neurofilaments, ganglia and nerve tracts were dramatically identified, including the pallial nerve entering the stellate ganglion and the 3^rd^ order giant fibers leaving the ganglion to innervate mantle muscles (Fig. 5D). No comparable staining pattern was observed in SG sections immunoreacted with FMRFamide antibodies.

## Discussion

The spatial and temporal characteristics of the expression of the *Fmrf* gene in the cephalopod *Loligo pealei* displayed several remarkable features that are relevant for the physiology of the tetrapeptide FMRFamide. Our study was prompted by the question as to whether the FMRFamide peptide, identified as a principal regulator of many molluskan neural functions, is a neuropeptide in the squid giant fiber system. Here, we compared the adult and hatchling giant fiber systems since the escape response mediated by this system's physiology differs at each of these stages (Otis and Gilly, 1990; Gilly et al., 1991). Glutamate, glycine and taurine have been indicated as neurotransmitters employed by the giant axon (Messenger, 1996; Gilbert et al., 1990). In an earlier study FMRFamide-immunoreactivity was detected in *Loligo pealei* SG extracts and was shown to potentiate transmission at the squid giant synapse by direct application of FLRF-amide to the SG (Cottrell et al., 1992). Although this study showed that SG neurons respond to a FMRF-related peptide, it remained uncertain if the peptide was also an endogenous transmitter of the efferent giant fiber system itself (Cottrell et al., 1992). The present finding of *Fmrf* cDNA as one of the most abundant cDNAs in EST libraries of the SG indeed suggested that FMRFamide might be an important neuropeptidergic transmitter of the giant axon. In addition, both *Fmrf* gene and peptide expression were reported in the developing SG of the cephalopod *Idiosepius notoides* (Wollesen et al., 2010). We therefore characterized *Fmrf* gene expression and gene products in adult and embryonic SGs in detail.

Despite high expression of the *Fmrf* gene in the adult GFL neurons that together form the giant fiber system, the results from proteomic and neuroanatomical analyses suggests that FMRFamide tetrapeptide is not an endproduct of *Fmrf* gene expression in the giant fiber system, as the content of FMRFamide tetrapeptide was unexpectedly low in extracts of the adult SG. The concentration of FMRFamide in SG extracts was at least 16-fold lower than in the optic lobe extracts, while the number of *Fmrf*-expressing neurons was higher in the SG than in the optic lobe (data not shown). Furthermore, FMRFamide immunostaining was virtually absent from the GFL neurons, which was consistent with the observation that no FMRFamide immunoreactivity was seen in the giant axons. In the SG, the strongest FMRFamide-immunoreactivity was found in small, scattered neurons in the ganglionic wall and fibers in the intraganglionic mass, rather than in the large neurons of the ganglionic walls. In the stellar nerves containing the 3^rd^ order giant axons as well as nerve fibers, FMRFamide-immunoreactivity was seen weakly in the smaller motor axons and more strongly in thin fibers surrounding the giant axon, but not in the giant axon. In the hatchling, FMRFamide was absent from the giant fiber system, while central brain lobes contained FMRFamide-expressing neurons and fiber tracts at this developmental stage. These results indicate that FMRFamide is not a major peptide product in the GFL and its giant fiber system, and therefore a role for FMRFamide tetrapeptide as neurotransmitter in the giant fiber system can be ruled out.

This study indicates that the FMRFamide tetrapeptide immunoreactivity detected in the adult SG is a product of the small intraganglionic neurons and is contained in fibers along the giant axon (Figs 2, 3). These small intraganglionic neurons have been described previously (Young, 1972), addressed as microneurons, and their function has been associated to reciprocal reflex actions through modulation of other neurons of the SG (Young, 1972). FMRFamide thus is a candidate neurotransmitter modulating such intraganglionic functions, in line with its potentiating effect of neurotransmission at the giant synapse (Cottrell et al., 1992). Additionally, FMRFamide may be contained in small fibers within the pallial nerve connecting the CNS to the peripheral SG, and it is present in fibers of the stellate neuropil. A similar distribution of FMRFamide immunoreactivity has been observed for *Sepia officinalis* (Aroua et al., 2011). FMRFamide may be an important neurotransmitter through which the CNS engages the SG in control of movement and respiration. The FMRFamide immunoreactive fibers from the pallial nerve associated with the giant axon may also belong to nerve fibers that pass through the SG without synapsing on the giant axon (Young, 1972). These may derive from the posterior chromatophore lobes that innervate the chromatophores in the epidermis of the mantle. Indeed, we have observed strong expression of *Fmrf* gene and peptide in the post-chromatophore lobe of the embryo and hatchling, which is consistent with this observation (data not shown). These neural projections may also contribute to some of the observed peptide expression within the neuropil fibers in both the adult and hatchling stellate ganglia.

The data reveal a notable mismatch between the high expression of the *Fmrf* gene in neurons of the GFL and the low content of FMRFamide immunoreactivity (Figs 2, 3). This shows translational regulation involving either inefficient *Fmrf* mRNA translation or, more likely, alternative precursor processing. We could not rule out one of these possibilities, since antisera to detect the precursor protein are lacking. It may well be that the main end product of FMRF precursor processing is not the FMRFamide tetrapeptide, but rather the intact or large fragments of the precursor protein escaping immunodetection. The proteomics technology was optimized for low molecular weight peptides and could not have detected such peptides if present. Tissue-specific differential precursor processing is a feature of many peptidergic systems in invertebrates and vertebrates, including mammals, and has physiological implications (Burbach, 2011b). A well-known example of differential precursor processing is proopiomelanocortin (POMC) that is fully processed to αMSH and βendorphin in hypothalamic neurons and the intermediate pituitary lobe to serve the modulation of various neural functions as neuropeptides, for instance in the regulation of feeding and energy homeostasis (Bicknell, 2008; Coll and Tung, 2009). In the anterior lobe of the pituitary gland POMC is only partially processed to ACTH and βLPH to serve peripheral hormonal functions. In some tumors of the human neuroectoderm POMC processing is often totally absent. The mechanisms of differential precursor processing involve the expression of different prohormone convertases (PCs) and the transport of newly synthesized precursor through different compartments of secretory pathways in which PCs are active (Pritchard and White, 2007; Hook et al., 2008; Seidah and Chrétien, 1999). These PCs may be investigated to explain the differential processing of the FMRF precursor in *Loligo pealei* when appropriate tools, like gene sequences, will become available.

The data rule out alternative splicing or expression of multiple *Fmrf* genes. One uniform *Fmrf* gene transcript (mRNA) was found in 228 cDNA clones, strongly suggesting that a single gene is expressed in the squid stellate ganglia and that alternative splicing does not occur in this tissue. Several other invertebrate species have multiple *Fmrf* genes. For example, *C. elegans* has as many as 23 FMRF-related genes (*flp-1* to *flp-23*; Kim and Li, 2004). Others, like the freshwater gastropod snail, *Lymnaea stagnalis*, express two distinct precursors by alternative splicing of one *Fmrf* gene transcript (Saunders et al., 1992). Both mechanisms are thought to enhance the diversity of FMRFamide-like peptides in order to serve a wide spectrum of physiological functions. In the SG of *Loligo pealei* the single *Fmrf* gene and single precursor should serve all required functions of FMRFamide, and perhaps alternative precursor processing is a way this species enhances FMRFamide diversity.

A second remarkable finding concerning *Fmrf* gene expression in the SG was the temporal regulation of gene expression. In the adult ganglion the level of *Fmrf* mRNA was high, both based on *in situ* hybridization and EST sequencing. In contrast, *Fmrf* mRNA could not be detected in the ganglion of late embryos and hatchlings, and FMRFamide immunoreactivity was absent in cell bodies of the ganglion. This suggests that *Fmrf* gene expression in the SG was induced only after hatching in the freely swimming squid during further development. It is tempting to speculate that engagement of stellate functions such as swimming and jet propulsion may be a factor involved in gene induction. This is quite possible since the electrophysiology of the escape behavior does exhibit a significant change at hatching in the squid *Loligo opalescens* (Gilly et al., 1991). Here, spontaneous rhythmic contractions of the mantle occur at stage 25 and an escape response can be elicited at stage 26 when stellar nerves contain small axons and no giant fiber. Vigorous escape jets can be induced in the absence of a giant axon, which makes its appearance only later at stage 28, after which vigorous jetting will occur. At hatching, latencies and synaptic delays of the escape response, declining during development, reach their adult levels (Gilly et al., 1991). Although this shift to an adult life may induce *Fmrf* gene expression and peptide synthesis in the ganglion, its contribution to the escape response seems only minor, perhaps as a modulator of the small motor axons. This contrasts with the developing CNS that does not display such a temporal induction of *Fmrf* gene expression between hatchling and adult in *Fmrf* gene expression. Intense *Fmrf* expression and FMRFamide production already exists in the embryonic brain lobes, nerve tracts and neuropils between stage 26 and 29 (data not shown). By hatching it appears that most if not all major sensory-motor networks underlying hatchling behavior have incorporated FMRFamide as a regulator of function, either as a neurotransmitter or a modulator of neural activation by other transmitters such as glutamate. Clearly, this suggested role for FMRFamide calls for an extensive electrophysiological analysis of neural pathways in the hatchling.

This study demonstrates that while FMRFamide is an abundant neuropeptide in the CNS of the squid *Loligo pealei*, it has a restricted role in the SG and efferent giant fiber system. Our findings indicate that *Fmrf* gene expression has adopted at least two mechanisms that render the SG FMRFamide system uniquely different from that of the CNS. Firstly, its expression is under strong developmental regulation in a tissue-specific fashion; it is suppressed in SG neurons in embryo and hatchling, but highly expressed in the adult SG neurons. Secondly, precursor processing may further generate biological activities that have functions of their own; FMRFamide tetrapeptide is the main product in the CNS, but minor in adult SG neurons. The biological significance of these mechanisms may well be to generate diversity in FMRF systems that is of physiological relevance in cephalopods.
